# Different Regulatory Effects of Heated Products and Maillard Reaction Products of Half-Fin Anchovy Hydrolysates on Intestinal Antioxidant Defense in Healthy Animals

**DOI:** 10.3390/ijms24032355

**Published:** 2023-01-25

**Authors:** Min Shi, Ru Song, Luo Gu

**Affiliations:** Key Laboratory of Health Risk Factors for Seafood of Zhejiang Province, School of Food Science and Pharmacy, Zhejiang Ocean University, Zhoushan 316022, China

**Keywords:** half-fin anchovy hydrolysates, thermal products, Maillard reaction products, intestinal antioxidant enzymes, oligopeptide transporter 1, Nε-carboxymethyllysine

## Abstract

The oxidative state of intestinal tracts of healthy animals were investigated after short-term intake of half-fin anchovy hydrolysates (HAHp) and their thermal or Maillard reaction products (MRPs). After one month of continuous oral gavage of HAHp, HAHp-heated products (HAHp-H), the MRPs of HAHp with 3% of glucose (HAHp-3%G MRPs), and the MRPs of HAHp with 3% of fructose (HAHp-3%F MRPs) at a dose of 1.0 g/kg of body weight per day into healthy ICR male mice, the concentrations of serum low-density and high-density lipoprotein cholesterol did not significantly change compared to the control group (CK, gavage with saline). Similar results were found for the interleukin-6 concentrations of all groups. By comparison, HAHp-H, HAHp-3%G MRPs, and HAHp-3%F MRPs administration decreased serum tumor necrosis factor-α concentration as compared to the CK group (*p* < 0.05). No histological damage was observed in the jejunum, ileum, and colonic tissues of all groups. However, HAHp-H treatment induced higher upregulation of Kelch-like ECH-associated protein 1, transcription factors Nrf-2, associated protective phase-II enzymes of NAD(P)H: quinine oxidoreductase-1, and hemoxygenase-1 in colon tissue, as well as higher upregulation of endogenous antioxidant enzymes, including copper/zinc superoxide dismutase, manganese superoxide dismutase, catalase, and glutathione peroxidase 2 than other groups (*p* < 0.05). Additionally, increases in Nε-carboxymethyllysine expression in the colonic tissues of all groups were consistent with their increased oligopeptide transporter 1 expressions. Our results suggest that the thermal products of HAHp might have a broad application prospect in improving antioxidant defense in vivo in healthy animals.

## 1. Introduction

The Maillard reaction (MR), also called the nonenzymatic browning reaction between the amino compounds of proteins, peptides, amino acids, and the carbonyl group of reducing carbohydrates [1], commonly occurs during food thermal processing or long-term storage [2]. In the MR process, hundreds of compounds are produced, including intermediates, volatile compounds and melanoidin, all of which are called Maillard reaction products (MRPs). Some documents have reported that MRPs-rich foods, such as bread and coffee, demonstrated excellent antioxidant and antibacterial activities [3,4]. In addition, MRPs usually have improved functionality and biological activity compared to their initial substrates of amino compounds or carbohydrates in MR [2], such as increases in the antibacterial activity of ε-polylysine/chitosan MRPs [5], the antioxidant activity of chitosan/glucose MRPs [6], glucose/lysine MRPs in preventing or treating inflammatory bowel disease [7], fish protein hydrolysates/ribose MRPs with enhanced emulsification and foaming capacities [8], etc. Therefore, there is a great interest in the utilization of MRPs as functional components in nutritional or health functions.

The health efficacy of the MRPs of peptides or protein hydrolysates have been extensively studied in different animal models. Chen, Fang, and Wang (2020) reported that, compared with fish scale peptides (FSP), the MRPs of FSP showed better liver protection against alcohol induced liver injury in mice by increasing the activities of hepatic superoxide dismutase (SOD), catalase (CAT), and glutathione peroxidase (GPX), and reducing the levels of hepatic malondialdehyde (MDA) and triglyceride (TG) [9]. Joung, Lee, Oh, and Kim (2021) found that stress-induced testicular dysfunction in mice was attenuated by MRPs from milk casein [10]. The administration of soybean peptide MRPs were found to alleviate aging-related disorders in D-galactose-induced ICR mice by reducing oxidative stress, systemic inflammation, and cognitive impairment of the brain [11]. However, there are limited reports on the effects of peptide or hydrolysate MRPs on the physiological functions of normal healthy animals.

Half-fin anchovy hydrolysates (HAHp), generated from the pepsin digestion of half-fin anchovy, contain peptides and amino acids [12]. In previous studies, we reported that the heated products of HAHp or its MRPs caused improved antioxidant, antibacterial, and anti-proliferative activities in vitro [13,14]. In addition, the intake of HAHp/glucose MRPs induced a higher antioxidant status in healthy mice after 14 days of oral gavage [15]. Furthermore, the administration of HAHp/glucose MRPs changed the composition of intestinal flora and correspondingly increased the contents of propionic acid and butyric acid in the feces of normal, healthy mice [16]. However, the regulatory effect of the heated products of HAHp or its MRPs on intestinal antioxidant defense in healthy animals remains unclear.

The aims of the present study were, therefore, (i) to investigate the different effects of HAHp’s heated products or MRPs intake on lipid oxidation and inflammation in healthy normal animals, (ii) to compare the histological changes in intestine tissues, and (iii) to evaluate the expression levels of the Kelch-like ECH-associated protein 1 (Keap1)/transcription factors Nrf-2 (Nrf2) pathway associated with protective phase-II enzymes of NAD(P)H: quinine oxidoreductase-1 (NQO-1) and hemoxygenase-1 (HO-1), as well as the activity of the endogenous enzymes SOD, CAT, and GPX in colonic mucosa, which were detected after 30 consecutive days of oral gavage into healthy mice. Our results were intended to reveal whether the heated products of HAHp or its MRPs have different regulatory effects on the intestinal antioxidant system of healthy normal animals, so as to provide a technical basis for the rational application of fish hydrolysates or peptide MRPs as functional ingredients in fortified foods.

## 2. Results and Discussion

### 2.1. Body Weight and Organ Index Measurement

After one month of oral gavage, none of the groups showed any significant differences in body weight as compared to the control group (CK) (*p* > 0.05) (Table 1). These results suggested that there were no obvious side effects of HAHp and its thermal or MRPs on the diet of mice.

No significant differences were found in the indexes of the heart, liver, spleen, or thymus in any of the tested groups as compared to the CK group (*p* > 0.05) (Table 2). However, decreases in kidney coefficient were found in all of the tested groups vs. the CK group. In particular, the HAHp (1.46 ± 0.14 g/100 g) and HAHp-3%F MRPs (1.52 ± 0.13 g/100 g) groups showed statistically lower kidney coefficients than the CK group (1.75 ± 0.18 g/100 g) (*p* < 0.05), which might suggest a certain degree of kidney atrophy, probably caused by HAHp and HAHp-3%F MRPs administration.

### 2.2. Serum Lipid and Inflammatory Factor Analyses

**Figure 1:**
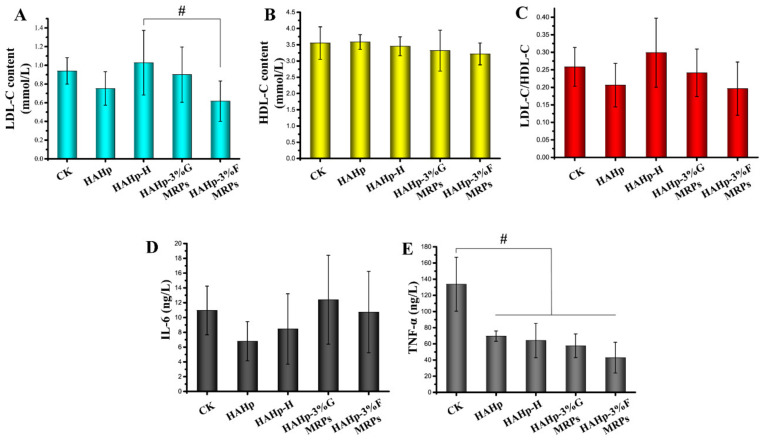
Effects of the oral administration of HAHp, HAHp-H, HAHp-3%G MRPs, and HAHp-3%F MRPs on serum lipid metabolism and inflammatory factors in mice after 30 days compared to theCK group. (**A**) Low density lipoprotein cholesterol (LDL-C) content, (**B**) high density lipoprotein cholesterol (HDL-C) content, (**C**) LDL-C/HDL-C ratio, (**D**) inflammatory factors interleukin-6 (IL-6) concentration, and (**E**) tumor necrosis factor-alpha (TNF-α) concentration. Results were expressed as the mean ± standard deviation (*n* = 7), and # represented 0.01 < *p* < 0.05.

In serum cholesterol transportation, low-density lipoprotein (LDL) plays a crucial role in oxidized LDL (ox-LDL) formation by reacting with reactive oxygen free radicals, and ox-LDL is considered to be a major risk factor for atherogenic disease [17,18]. Therefore, a high value of low-density lipoprotein cholesterol (LDL-C) in serum will cause a blood lipid metabolism disorder and induce various diseases, such as coronary heart disease and fatty liver [19]. In the present study, except for the HAHp-H group, all three of the other groups demonstrated a downward trend in serum LDL-C content as compared to the CK group (Figure 1A). The lowest LDL-C content (0.617 mmol/L) was detected in mice serum exposed to HAHp-3%F MRPs for one month compared with that in the HAHp-H group, which suggests the potential promotion effect of HAHp-3%F MRPs on blood lipid metabolism in mice.

Compared to LDLs, high-density lipoprotein (HDL) can act as a “scavenger” of lipids in serum by bringing extra cholesterol in peripheral tissues back to the liver for catabolism [20]. Therefore, HDL cholesterol (HDL-C) plays an important role in resisting and alleviating atherosclerosis [21]. After one month of oral administration, none of the groups showed any significant changes in the HDL-C content (Figure 1B) (*p* > 0.05). Similar to our result, Mastrocola et al. (2020) reported that mice exposed to an advanced glycation end products (AGE)-enriched diet for 22 weeks demonstrated similar HDL-C levels when compared to mice fed with a control diet [22].

Studies have shown that the LDL-C/HDL-C ratio is better suited to predict the situation of atherosclerosis and the degree of coronary artery disease than single lipid parameters such as LDL-C and HDL-C [23,24]. As shown in Figure 1C, the change in the LDL-C/HDL-C ratio was consistent with the LDL-C content change. Our results indicated the potential effects of HAHp and its MRP intake on improving blood lipid metabolism in mice to some extent.

In addition to body weight and metabolism-related LDL-C and HDL-C, other inflammatory parameters are commonly used to evaluate the immune regulation of the body after external substance stimulation, such as inflammatory factors interleukin-6 (IL-6), a proinflammatory cytokine produced by adipose tissue; and tumor necrosis factor-alpha (TNF-α), another pro-inflammatory cytokine produced mainly by macrophages [25,26]. IL-6 can influence the liver metabolism, and increases in IL-6 will significantly increase LDLs and hypertriglyceridemia levels [27]. TNF-α has been reported to be positively correlated with adaiposity, triglyceridemia, and insulin resistance, and negatively correlated with the HDL-C level [28]. Compared to the CK group, decreased IL-6 concentration trends were found in HAHp-, HAHp-H-, and HAHp-3%F MRPs-treated animals, although no significant differences were measured (Figure 1D). It was noted that dramatically lower TNF-α levels were observed in the HAHp, HAHp-H, HAHp-3%G MRPs, and HAHp-3%F MRPs groups compared to the CK (*p* < 0.05) (Figure 1E). These results indicated that the oral gavage of HAHp, HAHp-H, HAHp-3%G MRPs, and HAHp-3%F MRPs could contribute to increasing anti-inflammation in animals.

### 2.3. Histological Analysis of the Intestine Tract

**Figure 2:**
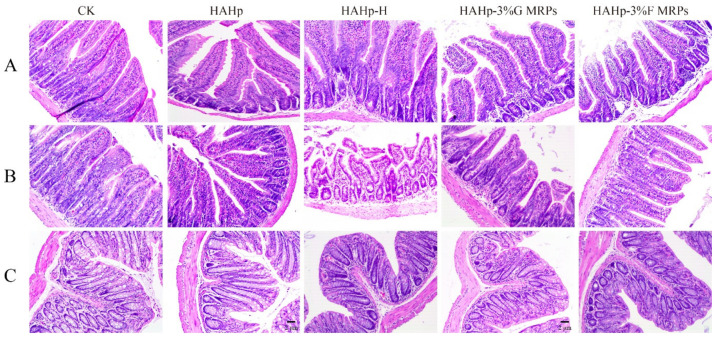
Histological analysis of intestine tracts after 30 days of oral gavage of HAHp, HAHp-H, HAHp-3%G MRPs, and HAHp-3%F MRPs compared to the CK (oral gavage of saline) by hematoxylin-eosin (H&E) staining. (**A**) Jejunum; (**B**) ileum, and (**C**) distal colon. Observation at 100× magnification.

In the CK group, the villi of jejunum and ileum were closely arranged and connected normally (Figure 2A,B). Similar observations were found in the HAHp group. In addition, the HAHp group had greater intestinal glands than the CK group. The intestinal glands can secrete intestinal juice containing various enzymes involved in food digestion. These digestive enzymes are attached to the free edge of the epithelium, which is the main place for nutrient absorption [29]. The changes in the intestinal glands of the HAHp group might be attributed to the enzyme secretion induced by HAHp administration. Similar phenomena involving greater intestinal glands were observed in the jejunum of HAHp-H-, HAHp-3%G MRPs-, and HAHp-3%F MRPs-treated groups. However, the microvilli in the HAHp-3%G MRPs group became slightly loose, and some villi were disturbed at the top in the HAHp-3%F MRPs-treated group. These results indicated that HAHp MRPs might induce changes in the normal tissue morphology of jejunum in mice to some extent after intragastric administration.

As for the ileum, increases of goblet cells were observed in the intestinal glands of the HAHp-H group or in the epithelium of the HAHp-3%F MRPs group compared to the CK group. Goblet cells can secrete a small amount of mucus with lubricating and protective effects [30]. The increased number of goblet cells in the HAHp-H and HAHp-3%F MRPs groups might be related to the fact that the HAHp-H and HAHp-3%F MRPs treatments did improve the body’s defense ability, or the spontaneous protective response of the intestinal tract in vivo to the invasion of external substances.

The colon is the largest microbial habitat in humans and animals, where undigested dietary components are digested, and then microbial metabolites can be absorbed by the colon mucosa [29]. Normal colonic tissues consist of mucosa, submucosa, muscularis, and adventitia. The colonic mucosa layer is thick and smooth with no villus. The epithelium of the colon is a monolayer columnar, composed of absorbing cells and goblet cells. The compact, straight tubular intestinal glands are distributed across the lamina propria [31]. After 30 days of oral gavage, the structures of colonic tissues in the CK group and the other four groups were complete and clear, the crypt structures were normal, and the goblet cells were scattered and not damaged. Furthermore, there was no congestion, erosion, and ulcer in the mucosa (Figure 2C).

**Figure 3:**
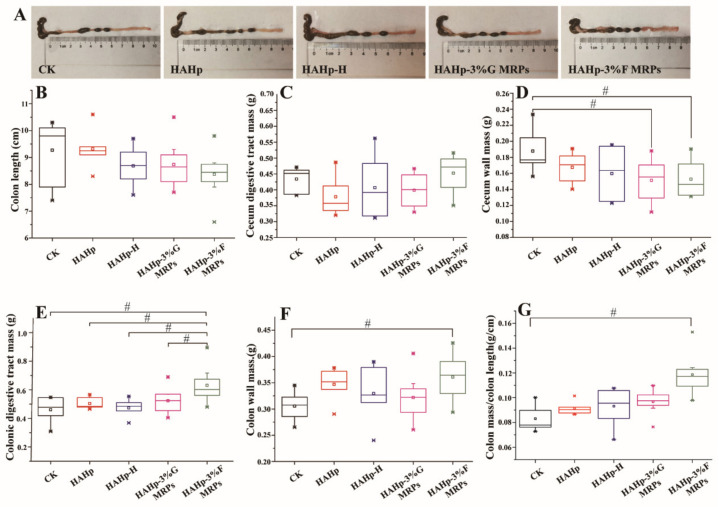
Cecum colonic intestinal environment of mice in the CK, HAHp, HAHp-H, HAHp-3%G MRPs, and HAHp-3%F MRPs groups after 30 days of oral gavage. (**A**) Intestinal photograph; (**B**) colon length, (**C**) cecum digestive tract mass; (**D**) cecum wall mass; (**E**) colonic digestive tract mass; (**F**) colon wall mass, and (**G**) ratio of colon mass/colon length. Data are expressed as the mean ± standard deviation (*n* = 7). # represents 0.01 < *p* < 0.05 vs. the CK group.

Colon length is an important indicator of colon growth, and changes in colon length are related to the efficiency of lipid and carbohydrate metabolism [32]. Colon shortening is considered to be a prominent feature of colitis in rodents [33]. The colon lengths of mice in the HAHp, HAHp-H, HAHp-3%G MRPs, and HAHp-3%F MRPs groups ranged from 8.5 cm to 9.0 cm, with no significant differences compared to the CK group (*p* > 0.05) (Figure 3A,B). Similar results were determined for the cecal digesta masses in all of the groups (*p* > 0.05) (Figure 3C).

Compared to the CK group, as shown in Figure 3D, the other four groups showed a downward trend in terms of cecum wall mass. Furthermore, HAHp-3%G MRPs and HAHp-3%F MRPs treatments decreased the cecal wall mass when compared to the CK (0.01 < *p* < 0.05). However, the HAHp-3%F MRPs group demonstrated higher colonic digestive tracts than the other groups (Figure 3E). Additionally, significant increases in colon wall mass were detected in the HAHp-3%F MRPs group as compared to the CK group (*p* < 0.05) (Figure 3F). The ratio of colon mass/colon length was higher in the HAHp-3%F MRPs group than that in the CK group (0.01 < *p* < 0.05) (Figure 3G). The results of Figure 3 suggest that, compared with other groups, HAHp-3%F MRPs could have a greater impact on the intestinal environment of the cecum and the colon.

### 2.4. Estimation of Cu/Zn-SOD (SOD1), Mn-SOD (SOD2), Catalase (CAT), Glutathione peroxidase 2 (GPX2), Oligopeptide Transporter 1 (PEPT1), and Nε-carboxymethyllysine (CML) Expression in Colonic Mucosa via Immunohistochemistry (IHC) Staining

**Figure 4:**
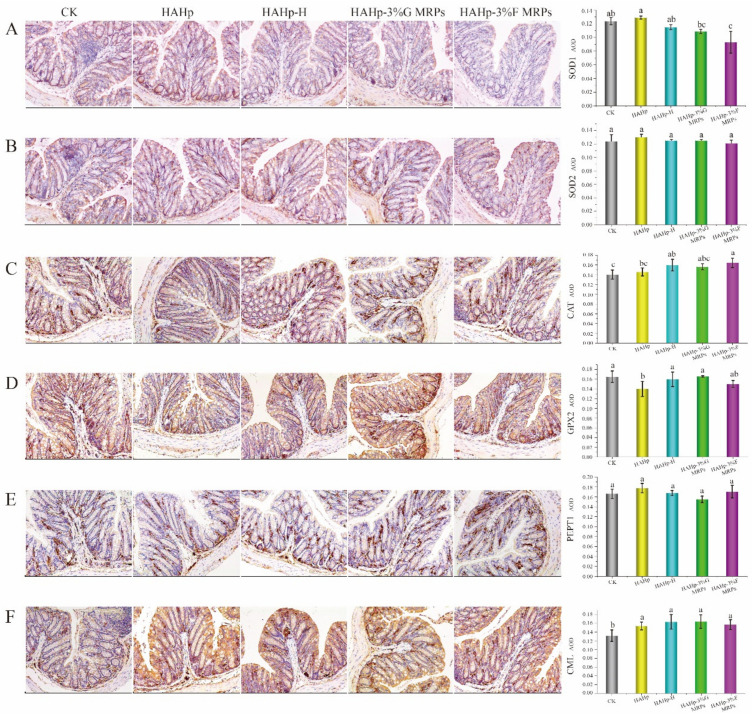
Protein expressions of Cu/Zn-SOD (SOD1), Mn-SOD (SOD2), catalase (CAT), glutathione peroxidase 2 (GPX2), oligopeptide transporter 1 (PEPT1), and Nε-carboxymethyllysine (CML) found *via* immunohistochemistry (IHC) (100×) analysis of the colon tissues of mice after 30 days of oral administration of HAHp, HAHp-H, HAHp-3%G MRPs, and HAHp-3%F MRPs compared to the CK. (**A**) SOD1, (**B**) SOD2, (**C**) CAT, (**D**) GPX2, (**E**) PEPT1, and (**F**) CML. In average optical density (AOD) analysis, different lowercase letters represent significant differences (*p* < 0.05).

The activities of key endogenous enzymes, such as SOD, CAT, and glutathione peroxidase 2 (GPX2), are associated with oxidative stress [15]. Positive expression of these enzymes will be indicated by brown stains during immunohistochemistry (IHC) staining. Cu/Zn-SOD (SOD1) and Mn-SOD (SOD2) are the two major isoforms of SOD in animals. The former exists in the cytoplasm of blood cells, and the latter is distributed in the mitochondrial matrix [34,35]. After one month of oral administration, SOD1 expression in the HAHp-3%F MRPs group was downregulated as compared to the CK group and as compared to the HAHp and HAHp-H groups (*p* < 0.05) (Figure 4A). By comparison, no significant changes were observed for the SOD2 expression in any of the groups (*p* > 0.05) (Figure 4B). SOD1 plays a crucial role in maintaining reactive oxygen species (ROS) homeostasis in cells. Abnormality of SOD1 is considered to be associated with some diseases, such as Down’s syndrome [36], cancer [37], amyotrophic lateral sclerosis [38], etc. Therefore, decreases in SOD1 expression in the HAHp-3%F MRPs group indicated that the absorption of HAHp-3%F MRPs might cause side effects on health.

As a central antioxidant-related enzyme in defending cells against oxidative stress, CAT can convert H_2_O_2_ into water and oxygen [39]. Furthermore, CAT is considered to be the only antioxidant enzyme that can be strongly glycated in vivo [40]; therefore, it is usually used as an indicator enzyme for glycation in vivo. As was shown in Figure 4C, all of the groups showed positive CAT expression in the colonic epithelial cells, intestinal glands, and submucosa, among which, the HAHp-H and HAHp-3%F MRPs groups showed stronger positive expressions than the CK group. Moreover, higher average optical density (AOD) was determined in the HAHp-3%F MRPs group compared to the HAHp group (*p* < 0.05). The IHC results for CAT showed that HAHp-H and HAHp-3%F MRPs treatments contributed to increases in the CAT activity in the colon of mice.

GPX2 has the function of reducing toxic peroxides to non-toxic hydroxyl compounds [39]. Similar to the CAT results, all of the groups had positive GPX2 expressions in the epithelial cells, intestinal glands, and submucosa (Figure 4D). However, compared to the HAHp group, higher positive areas were detected in the HAHp-H and HAHp-3%G MRPs groups (*p* < 0.05), suggesting that they have stronger effects on the activity of GPX2 increases.

Oligopeptide Transporter 1 (PEPT1), a dipeptide/tripeptide transporter, is usually expressed in the brush border membrane of villous intestinal absorption epithelial cells [41], which physiologically facilitates the absorption of dipeptide/tripeptide nutrients and peptidomimetic drugs [42]. In recent years, it has been found that PEPT1 is critical to intestinal homeostasis in terms of metabolite characteristics and histophysiology, and dysfunction of PEPT1 always leads to intestinal diseases [43]. At the present study, it was worth noting that PEPT1-positive expression regions were detected in the lamina propria and submucosa of all groups (Figure 4E), although there were no significant differences in the AOD results among these groups (*p* > 0.05).

Nε-carboxymethyllysine (CML) is a common AGE formed both in heated foods and the human body [44]. AGE-enriched diet fed can evoke CML accumulation in the villi epithelium of the ileum of mice compared to the control mice [22]. In this study, compared with the CK group, CML was strongly expressed in the epithelial cells of the HAHp, HAHp-H, HAHp-3%G MRPs, and HAHp-3%F MRPs groups (*p* < 0.05). However, no significant differences were measured in the AOD results between the HAHp group and the HAHp-H or HAHp MRPs groups (*p* > 0.05). Our findings implied the accumulation of CML in the colonic tissues after intake of HAHp and its thermal products or MRPs.

### 2.5. Real-Time Quantitative Polymerase Chain Reaction (qRT-PCR) Analysis of Colonic Mucosa

To further investigate the effects of HAHp and its related MRPs on the antioxidant enzymes and active factors related with oxidative stress in the colon, the mRNA expression gene levels of *Keap1, Nrf2, NQO1, HO-1, SOD1, SOD2, CAT, GPX2, PEPT1*, and *CML* were compared.

**Figure 5:**
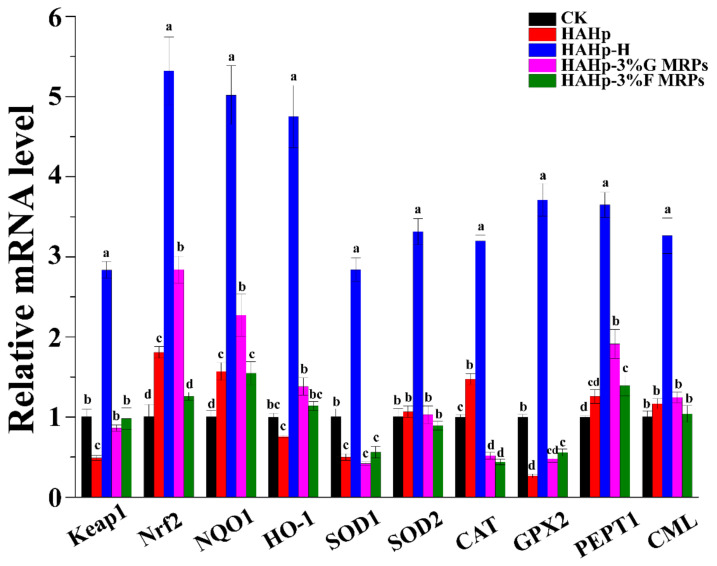
Effects of HAHp, HAHp-H, HAHp-3%G MRPs, and HAHp-3%F MRPs treatments on the related mRNA expression levels of Kelch-like ECH-associated protein 1 (*Keap1*); transcription factors Nrf-2 (*Nrf2*); genes associated protective phase-II enzymes NAD(P)H: quinine oxidoreductase-1 (*NQO-1*) and hemoxygenase-1 (*HO-1*); genes of *SOD1, SOD2, CAT*, and *GPX2* associated with the antioxidant enzymes; *PEPT*; and *CML* in mice colon mucosa after 30 days oral administration. Different lowercase letters (a–d) in the same index represent significant difference (*p* < 0.05).

Under normal conditions, Nrf2 in cytoplasm combines with its inhibitor, Keap1. However, some stressful conditions that occur in vivo, such as intracellular ROS accumulation, will disrupt the Keap1/Nrf2 interaction by oxidizing key cysteine residues in Keap1, which results in Nrf2 dissociating from Keap1 [45]. Subsequently, released Nrf2 can translocate into the nucleus to stimulate antioxidant responsive element (ARE), which will regulate the expression of many antioxidants and cytoprotective genes by binding to ARE motifs [46]. Therefore, the Keap1/Nrf2 pathway is one of the most important endogenous protective mechanisms involved in the modulation of oxidative stress [46]. As was shown in Figure 5, increases in *Keap1* and *Nrf2* mRNA levels in the HAHp-H group suggested a typical Keap1/Nrf2 pathway involved in its antioxidant system. By comparison, the oral administration of HAHp and HAHp-3%G MRPs greatly activated expression of the *Nrf2* gene, whereas dramatic increases in *Keap1* mRNA level were not detected compared to the CK group. As a downstream gene of *Nrf2*, the enhanced *NQO1* expression can protect cells when Nrf2 is activated in large amounts [47].

The upregulated mRNA levels in the Nrf2-associated enzyme gene of *NQO1* were found in the HAHp-, HAHp-H-, HAHp-3%G MRPs-, and HAHp-3%F MRPs-treated groups, indicating their potential effect with regard to increasing antioxidant activity in mice by activating the expression of the Nrf2-regulated phase II enzyme, NQO1. Furthermore, a stronger mRNA level for *HO-1*, another Nrf2-associated enzyme, was measured in the HAHp-H group. Similarly, the mRNA levels of *SOD1*, *SOD2*, *CAT*, and *GPX2* were increased significantly in the HAHp-H group as compared to the other groups (*p* < 0.05). The above results indicated the stronger facilitating effect of HAHp-H on the transcription of genes in the Keap1/Nrf2 pathway.

In the HAHp, HAHp-H, HAHp-3%G MRPs, and HAHp-3%F MRPs groups, the relative expressions of the *PEPT1* gene were significantly upregulated compared with the CK group (*p* < 0.05). Among them, the HAHp-H group showed the largest expression of the *PEPT1* gene, indicating its stronger role in activating the *PEPT* gene in the colon mucosa. The inconsistency between the *PEPT1* mRNA level and IHC result (as shown in Figure 4E) might be related to the fact that the total RNA extracted from the colon mucosa might contain intestinal flora RNA, resulting in the upregulation of *PEPT1* expression in qRT-PCR analysis. Compared with the groups administered with HAHp or its MRPs, the enhanced gene expression of *CML* in the HAHp-H group also suggested the stronger function of HAHp-H in activating CML accumulation in the colon.

### 2.6. Western Blot (WB) Analysis of Colonic Mucosa

**Figure 6:**
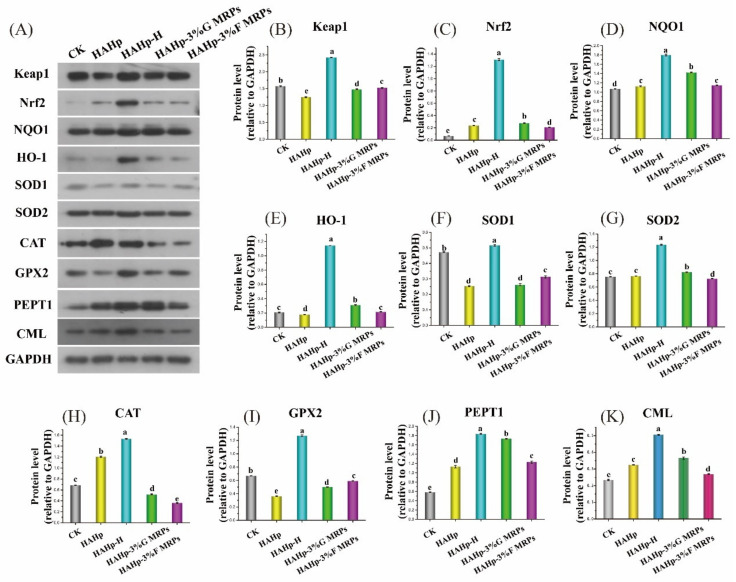
Effects of HAHp, HAHp-H, HAHp-3%G MRP, and HAHp-3%F MRP treatments on the expression of Keap1, transcription factors Nrf2, associated protective phase-II enzymes of NQO-1 and HO-1, related antioxidant enzymes (SOD1, SOD2, CAT, GPX2), PEPT1, and CML proteins relative to the glyceraldehyde-3-phosphate dehydrogenase (GAPDH) in the colonic mucosas of mice measured *via* western blot (WB) analysis after 30 days administration compared to the CK. (**A**) WB bands, (**B**) Keap1 protein level, (**C**) Nrf2 protein level, (**D**) NQO-1 protein level, (**E**) HO-1 protein level, (**F**) SOD1 protein level, (**G**) SOD2 protein level, (**H**) CAT protein level, (**I**) GPX2 protein level, (**J**) PEPT1 protein level, and (**K**) CML level. In (**B**–**K**), data were presented as the mean ± standard deviation (*n* = 3). Different lowercase letters (a–e) represent significant difference (*p* < 0.05).

Similar to the mRNA expression results, the relative protein expression of Keap1 was significantly upregulated after HAHp-H treatment compared to the other groups (*p* < 0.05). Increases in Nrf2 protein expression in the HAHp, HAHp-H, HAHp-3%G MRPs, and HAHp-3%F MRPs groups confirmed the dissociation of Nrf2 from Nrf2-Keap1 conjugate, which could active the expression of anti-oxidative enzymes. As expected, higher NQO1 protein expressions were detected in the HAHp-, HAHp-H-, HAHp-3%G MRPs-, and HAHp-3%Fs MRP-treated groups as compared to the CK group (*p* < 0.05). In addition to an increase in the NQO1 protein level, the HAHp-H-treated group showed higher protein expressions of HO-1, SOD1, SOD2, CAT, and GPX2 than the other groups, although HAHp-3%G MRPs treatment upregulated higher HO-1 and SOD2 protein expressions than the CK group (*p* < 0.05), and HAHp increased the CAT protein level compared to the CK group (*p* < 0.05) (Figure 6E–I). These results suggest that HAHp-H feed can promote the antioxidation of the colon in mice.

It is noted that the relative protein expressions of SOD1, CAT, and GPX2 were downregulated significantly in the HAHp-3%G MRPs and HAHp-3%F MRPs groups compared to the CK group (*p* < 0.05) (Figure 6F,H,I). This indicated differences in the antioxidative defenses of the murine colon, induced by treatment using HAHp thermal products and its MRPs. As an indicator of antioxidant enzyme strongly glycated in vivo [40], the decreases in CAT protein expression in the HAHp-3%G MRPs and HAHp-3%F MRPs groups (Figure 6H) suggested their stronger glycation influences on colon than HAHp or its thermal products. Decreases in GPX2 protein expressions determined in the HAHp, HAHp-3%G MRPs, and HAHp-3%F MRPs groups (Figure 6I) were consistent with their mRNA expression trends shown in Figure 5.

Compared to the CK group, the protein expression level of PEPT1 was upregulated significantly in the other four groups (*p* < 0.05) (Figure 6J). Microbial fermentation in the colon may be related to the enhanced PEPT1 expression, which also suggested that peptide could be further digested and utilized by colonic microorganisms [48].

CML is a considered to be an indicator of AGE formation in food [49]. In this study, increases in CML expression in these groups were detected in western blot (WB) results as compared to the CK group (Figure 6K). Furthermore, HAHp-H treatment induced stronger CML expression than HAHp or its MRPs. Some animal experiments have confirmed that the administration of CML, either in the free state (glycated with free amino acids) or in the bound state (covalently conjugated with peptides/proteins) [50,51], could induce the accumulation of total CML in the colon and ileum [52]. Recently, Yuan et al. (2022) revealed that the colon was the main tissue for the accumulation of free and bound CML [53]. Interestingly, our findings revealed that the increased colonic CML expression levels in these groups were consistent with their PEPT1 expression levels. Free CML are likely to be absorbed into intestinal cells by simple diffusion. By comparison, bound CML in the form of di-tripeptides or polypeptides could most probably be absorbed by the peptide transporter PEPT1 or endocytosis [54]. Therefore, increases in PEPT1 and CML expressions, as were shown in Figure 6J,K, might be ascribed to unabsorbed bound CML partly retained directly in the intestinal cells, which could further elicit PEPT1 and CML expressions in the colon. In addition, these findings suggested that a fraction of ingested CML, which were not absorbed, might enter the colon and be metabolized by the colonic microbiota.

## 3. Materials and Methods

### 3.1. Materials

Half-fin anchovy (*Setipinna taty*) was purchased from a local aquatic market in Zhoushan, China. Pepsin (1200 U/g) was obtained from Sinopharm Chemical Reagent Co., Ltd. (Shanghai, China). The assay kits of LDL-C, HDL-C, IL-6, and TNF-α were purchased from Jiancheng Bioengineering Institute (Nanjing, China). Primary antibodies against SOD1, SOD2, CAT, GPX2, Nrf2, and CML were purchased from Abclonal (Wuhan, China). Primary antibodies against Keap1, NQO-1, and HO-1 were bought from Abcam (Shanghai, China). A primary antibody against PEPT1 was purchased from Santa Cruz Biotechnology, Inc. (Beijing, China). A primary antibody against GAPDH was purchased from Sigma (St Louis, MO, USA). An HRP-labeled goat anti-rabbit/mouse IgG (PV-9000) universal two-step kit was obtained from Beijing Zhongshan Jinqiao Biotechnology Co., Ltd. China. Other chemicals of analytical grade were purchased from Sinopharm Chemical Reagent Co., Ltd. (Shanghai, China).

### 3.2. Preparation of Half-fin Anchovy Hydrolysates (HAHp) and Its Thermal Products and Maillard Reaction Products (MRPs)

HAHp was prepared with reference to the optimum method used in our previous study [12]. Briefly, the minced fish muscle was mixed with deionized water at a ratio of 1:4 (*w*/*v*) with 1100 U/g of pepsin addition and hydrolyzed for 2.4 h at 37 °C. Then, the mixture was boiled at 95 °C for 10 min to deactivate the pepsin. After pH adjustment to 7.0, the mixture was centrifuged at 7000× *g* for 20 min (4 °C), and the collected supernatant was named HAHp. Then, the HAHp was heated at 120 °C for 30 min by an autoclave sterilizer (DSX-280KB24, Shenan Medical Equipment Factory, Shanghai, China) to simulate food thermal sterilization, and the generated thermal products were named HAHp-H and stored at −20 °C for further use.

The MRPs of HAHp with glucose or fructose were prepared by heating the mixture at a controlled temperature of 120 °C in an oil bath (ZNCL-GS130×70, Hangzhou Gengyu Instrument Co., Ltd., Hangzhou, China) as follows: glucose or fructose was added to HAHp to obtain a sugar concentration of 3%(*w*/*v*). Once completely dissolved, the mixture solution was heated either for 90 min to prepare the MRPs of HAHp and glucose, or for 120 min to prepare the MRPs of HAHp and fructose, (based on the results of Figures S1 and S2). The generated MRPs HAHp/glucose and HAHp/fructose were named HAHp-3%G MRPs and HAHp-3%F MRPs, respectively, and kept at −20 °C until use.

### 3.3. Animals and Experiment Design

ICR mice (male, weight 18–22 g, age 4 weeks old) were purchased from Hangzhou Ziyuan Experimental Animal Technology Co., Ltd., China, with a Zhejiang Experimental Animal Quality Certification (No. 20211025Abzz0105999312). All animals were acclimatized in house at 22 ± 2 °C, with 50 ± 10% relative humidity and 12/12 h light/dark automatically. After a week of acclimatization, during which they were fed with a standard chow diet and provided water ad libitum, all animals were randomly divided into five groups, with seven to eight animals in each group—including the control (CK, oral gavage with saline), HAHp, HAHp-H, HAHp-3%G MRPs, and HAHp-3%F MRPs groups—at a dose of 1.0 g per kg per day bw for 30 consecutive days. The experiment was performed according to the Guide for the Care and Use of Laboratory Animals (National Research Council, 2016) and approved by the Ethics Committee of Experimental Animal Care of Zhejiang Ocean University (2021064).

### 3.4. Blood and Tissues Collection

All groups of animals fasted for 24 h at the end of the last gavage. The blood of the mice was collected under ether anesthesia, centrifuged at 3000× *g* for 15 min at 4 °C to obtain serum, and stored at −80 °C until use. The tissues of the heart, liver, spleen, kidney, and thymus were collected. After measuring the length of the colon, about a 1 cm length was cut from the distal colon and fixed with 4% paraformaldehyde. The remaining colon mucosa was collected in an ice bath and stored at −80 °C until use.

### 3.5. Body Weight and Organ Index Measurement

The mice were weighed weekly before oral gavage, and the last weights were measured before all of mice were killed. After the removal of excess fat and blood, the weights of the heart, liver, spleen, kidney, and thymus of all animals were determined, and the organ indexes were calculated as follows:

 Heart coefficient (mg/g)=[Heart weight (mg)/Mouse weight(g)] 

 Liver coefficient (g/100 g)=[Liver weight (g)/Mouse weight(g)]×100

Spleen coefficient (mg/g)=[Spleen weight (mg)/Mouse weight(g)] 

Kidney coefficient (g/100 g)=[Kidney weight (g)/Mouse weight(g)]×100

Thymus coefficient (mg/g)=[Thymus weight (mg)/Mouse weight(g)] 

### 3.6. Lipid Oxidation and Proinflammatory Cytokines Measurement

LDL-C and HDL-C related to lipid oxidation and two typical proinflammatory cytokines, IL-6 and TNF-α, were measured according to the instructions of the corresponding assay kits.

### 3.7. Histopathology

After being fixed with 4% paraformaldehyde overnight, the intestine tissues, including the jejunum, ileum, and colon, were embedded in paraffin, cut into 5 μm-thick sections, de-waxed, hydrated, and stained using hematoxylin-eosin (H&E). The histopathologic changes of intestine tissues were observed through an optical microscope (E100/E100LED MV, Nikon, Japan) under 100 magnifications.

### 3.8. IHC Measurement

IHC was performed to estimate the levels of SOD1, SOD2, CAT, and GPX2 related with endogenous antioxidant enzyme activities, and PEPT1 and CML associated with peptides and MRPs accumulation in colonic tissues. In brief, paraffin sections of the colon were placed on poly-l-lysine-coated slides, deparaffinised in xylene, hydrated with graded alcohol for 3 min each time, and, finally, rinsed with PBS three times. Then, the sections were repaired with 0.01M of sodium citrate buffer (pH 6.0) antigen repair solution for 20 min at 100 °C. Next, they were washed twice with PBST and once with PBS, for 5 min each time. After being incubated with 3% H2O2 solution at room temperature for 10 min and rinsed three times with PBS, all of the sections were blocked in 1–2% goat serum at room temperature for 30 min. After the removal of the blocking solution, 50 μL of the primary antibodies at 1:500 dilutions, including anti-SOD1, anti-SOD2, anti-CAT, anti-GPX2, anti-PEPT1, and anti-CML, were respectively added into the slices and incubated overnight at 4 °C. This was followed by PBS rinsing three times for 5 min each time. Then, polymer auxiliary agent 1 was added, and the sections were incubated at 37 °C for 20 min. After incubation with the secondary antibody (HRP labeled goat anti rabbit/mouse IgG 1: 500) for 2 h at room temperature, the sections were washed with PBS three times for 5 min each time and stained with chromogenic reagent 3, 3′-diaminobenzidene (DAB) for 30 s. After being counterstained with hematoxylin for 5 min, dehydrated, cleared in xylene, and fixed in mounting media, all of the slides were observed using an optical microscope (E100/E100LED MV, Nikon, Japan). With reference to the CK group, the positive degrees of tested protein in colonic sections were digitalized as mean integrated optical density (IOD) using the image analysis software Image Pro Plus 6.0 (Media Cybernetics, Rockville, MD, USA).

### 3.9. qRT-PCR Analysis

Using *GAPDH* as an internal reference, the result of 2^−ΔΔCt^ was used to determine the relative mRNA expression levels of target genes in the colonic mucosa. The total RNA was extracted with Trizol reagent, and the isolated total RNA (OD260/280 = 1.8~2.2) was quantified with a MX3000P real-time fluorescence quantitative PCR instrument (Stratagene, U.S.). The details of the qRT-PCR primer sequences were shown in Table 3.

The qRT-PCR cycling conditions were 94 °C for 3 min, followed by 40 cycles of 94 °C for 10 s and 60 °C for 40 s. All of the samples were carried out in triplicate, and the results were calculated as the 2^−ΔΔCt^ value normalized to the *GAPDH* gene level of the CK group.

### 3.10. Estimated Expressions of Related Proteins by WB

The expression levels of Keap1, Nrf2, NQO1, HO-1, SOD1, SOD2, CAT, GPX2, PEPT1, and CML in the colonic mucosa were evaluated by WB analysis [55,56] with slight modifications. In brief, the intestinal mucosa was blended with 300 μL of RIPA cell lysate to lyse the cells. Next, 3 μL samples were blended with 10 μL of 2 × sodium dodecyl sulfate-polyacrylamide gel electrophoresis (SDS-PAGE) loading buffer, heated for 5 min under 100 °C, cooled on ice, and centrifuged at 12,000× *g* for 5 min to remove insoluble precipitates. Then, 20 μL of supernatant was separated via 10% SDS-PAGE and transferred onto a polyvinylidene difluoride membrane by semi dry electrophoretic transfer under 30 mA for 60 min, and blocked using a blocking buffer overnight at 4 °C. Subsequently, the membrane was washed with 1 × TBST three times for 15 min each. Then, the membrane was incubated with specific anti-Keap1 (rabbit polyclonal 1:1000), anti-Nrf2 (rabbit polyclonal 1:1000), anti-NQO-1 (rabbit polyclonal 1:2000,), anti-HO-1 (rabbit polyclonal 1:2000), anti-SOD1 (rabbit polyclonal 1:4000), anti-SOD2 (rabbit polyclonal 1:1000), anti-CAT (rabbit polyclonal 1:2000), anti-GPX2 (rabbit polyclonal 1:1000), anti-PEPT1 (rabbit polyclonal 1:200), anti-CML (rabbit polyclonal 1:1000) or anti-GAPDH (rabbit polyclonal 1:5000) at 37 °C for 2 h, and washed with 1 × TBST four times for 10 min each. After incubation with HRP-conjugated goat anti-mouse IgG (1:1000) at 37 °C for 2 h, the membrane was again washed with 1 × TBST four times for 10 min each. Chemiluminescence detection was carried out with Super-GL ECL supersensitive luminescence liquid, and the X-ray film was exposed. After developing and fixing, the dried film was finally photographed using the Bio Spectrum Gel Imaging System (UVP, USA). Gel-Pro Analyzer software was used to analysis the results, and the expression level of the objective protein was expressed as the IOD of the objective protein versus the IOD of GAPDH.

### 3.11. Statistical Analysis

Data were expressed as mean ± standard deviation. The statistical differences among the groups were analyzed using one-way analysis of variance (ANOVA) with Duncan’s multiple range test, and *p* < 0.05 was declared significant. All statistical analyses were performed using the SPSS^®^ software (SPSS Statistical Software 22.0, Inc., Chicago, IL, USA).

## 4. Conclusions

Our results confirmed the different regulatory effects of HAHp, HAHp-H, HAHp-3%G MRPs, and HAHp-3%F MRPs on intestinal antioxidant defense in healthy animals. The administration of HAHp, HAHp-H, HAHp-3%G MRPs, and HAHp-3%F MRPs can significantly decrease the TNF-α level. HAHp-H and HAHp-3%G MRPs treatments increased the GPX2 expression in IHC results. The higher CML expression in the groups treated with HAHp and its thermal products or MRPs as compared to the CK group in the results of IHC suggested CML accumulation and further absorption in the colon tissue. However, compared with the expression levels of Keap1, Nrf2, NQO1, HO-1, SOD1, SOD2, CAT, GPX2, and PEPT1 in all of the groups based on the results of qRT-PCR and WB, it was obvious that HAHp-H demonstrated the strongest effect of the groups in increasing the intestinal antioxidant activity in healthy animals. However, further investigations are needed to implore the enter-pathway of CML in colon tissue, and thereby how to affect the balance of colon flora.

## Figures and Tables

**Table 1:** Changes in body weight after oral gavage with HAHp, HAHp-H, HAHp-3%G MRPs, and HAHp-3%F MRPs for 30 days compared to the CK group (*n* = 7 per group).

Group	0 d	7 d	14 d	21 d	28 d	30 d
CK	28.69 ± 1.75 ^a^	29.74 ± 2.97 ^a^	31.42 ± 3.06 ^a^	33.80 ± 2.82 ^a^	35.31 ± 2.76 ^ab^	36.00 ± 3.07 ^a^
HAHp	28.35 ± 0.92 ^a^	29.75 ± 1.19 ^a^	31.30 ± 1.23 ^a^	33.05 ± 1.41 ^a^	34.01 ± 1.45 ^b^	34.88 ± 1.56 ^a^
HAHp-H	28.11 ± 0.97 ^a^	30.34 ± 1.46 ^a^	32.73 ± 1.91 ^a^	34.46 ± 1.88 ^a^	35.76 ± 2.3 ^ab^	36.43 ± 2.32 ^a^
HAHp-3%G MRPs	29.29 ± 0.83 ^a^	31.73 ± 1.53 ^a^	33.84 ± 1.42 ^a^	35.48 ± 1.42 ^a^	36.94 ± 1.8 ^a^	37.49 ± 1.88 ^a^
HAHp-3%F MRPs	28.43 ± 1.15 ^a^	31.03 ± 1.37 ^a^	32.60 ± 1.90 ^a^	34.31 ± 1.77 ^a^	35.80 ± 2.06 ^ab^	36.23 ± 2.12 ^a^

CK: oral gavage with saline; HAHp: oral gavage with half-fin anchovy hydrolyses; HAHp-H: oral gavage with heated HAHp; HAHp-3%G MRPs: oral gavage with the MRPs of HAHp and glucose (3%); HAHp-3%F MRPs: oral gavage with the MRPs of HAHp and fructose (3%). Different lowercase letters in the same column represent significant differences (*p* < 0.05) in body weight between different groups.

**Table 2:** Organ indices of the heart, liver, spleen, kidney, and thymus compared among all of the tested groups after 30 days of oral gavage (*n* = 7 per group).

Group	Heart (mg/g)	Liver (g/100 g)	Spleen (mg/g)	Kidney (g/100 g)	Thymus (mg/g)
CK	5.55 ± 0.82 ^a^	4.48 ± 0.20 ^a^	2.76 ± 0.54 ^a^	1.75 ± 0.18 ^a^	1.82 ± 0.70 ^a^
HAHp	5.48 ± 0.67 ^a^	4.47 ± 0.51 ^a^	2.44 ± 0.63 ^a^	1.46 ± 0.14 ^c^	1.75 ± 0.54 ^a^
HAHp-H	5.26 ± 0.46 ^a^	4.34 ± 0.51 ^a^	2.77 ± 0.37 ^a^	1.68 ± 0.11 ^ab^	1.66 ± 0.36 ^a^
HAHp-3%G MRPs	5.16 ± 0.47 ^a^	4.55 ± 0.30 ^a^	2.53 ± 0.35 ^a^	1.59 ± 0.14 ^abc^	1.92 ± 0.45 ^a^
HAHp-3%F MRPs	5.11 ± 0.39 ^a^	4.28 ± 0.17 ^a^	2.73 ± 0.58 ^a^	1.52 ± 0.13 ^bc^	2.08 ± 0.50 ^a^

Different lowercase letters in the same column indicate significant difference (*p* < 0.05).

**Table 3:** Synthesis sequence of qRT-PCR primers.

Genes	Upper Primers (5’, 3’)	Downstream Primers (5’, 3’)	bp
*Keap1*	AATGTTGACACGGAGGATTGG	ATCCGCCACTCATTCCTCTC	132
*Nrf2*	CTTCCATTTACGGAGACCCAC	GATTCACGCATAGGAGCACTG	175
*NQO1*	TCAACTGGTTTACAGCATTGGC	GCTTGGAGCAAAATAGAGTGGG	118
*HO-1*	CGAATGAACACTCTGGAGATGAC	GCCTCTGACGAAGTGACGC	166
*SOD1*	GTGACTGCTGGAAAGGACGG	CAATCCCAATCACTCCACAGG	196
*SOD2*	GAGGCTATCAAGCGTGACTTTG	GCAATGGGTCCTGATTAGAGC	157
*CAT*	ATTGCCGTTCGATTCTCCAC	TCCCACAAGATCCCAGTTACC	117
*GPX2*	CCTGGATGGGGAGAAGATAGAC	CGAACTGGTTGCAAGGGAAG	170
*PEPT1*	GGCTTCTAACTGTGGCGGTC	CTCTGCTGGGTTGATGTAGGTG	167
*CML*	TCCTTCACGACTTGCTAAAACAC	TCCTCGTTGATGCTGGACAG	106
*GAPDH*	TGTTCCTACCCCCAATGTGTC	TGAAGTCGCAGGAGACAACC	158

## Data Availability

All data presented in the manuscript is available upon request.

## Supplementary Materials

The following supporting information can be downloaded at: https://www.mdpi.com/article/10.3390/ijms24032355/s1.

## Abbreviations

HAHp	Half-fin anchovy hydrolysates
MR	Maillard reaction
MRPs	Maillard reaction products
HAHp-H	Heated products of HAHp
HAHp-3%G MRPs	MRPs of HAHp with 3% of glucose
HAHp-3%F MRPs	MRPs of HAHp with 3% of fructose
FSP	Fish scale peptides
SOD	Superoxide dismutase
CAT	Catalase
GPX	Glutathione peroxidase
MDA	Malondialdehyde
TG	Triglyceride
Keap1	Kelch-like ECH-associated protein 1
Nrf2	Transcription factors Nrf-2
NQO-1	NAD(P)H: quinine oxidoreductase 1
HO-1	Hemoxygenase-1
LDL	Low-density lipoprotein
ox-LDL	Oxidized low-density lipoprotein
LDL-C	Low-density lipoprotein cholesterol
HDL	High-density lipoprotein
HDL-C	High-density lipoprotein cholesterol
AGE	Advanced glycation end products
IL-6	Inflammatory factors interleukin-6
TNF-α	Tumor necrosis factor-alpha
H&E	Hematoxylin-eosin
IHC	Immunohistochemistry
SOD1	Copper/zinc superoxide dismutase
SOD2	Manganese superoxide dismutase
ROS	Reactive oxygen species
AOD	Average optical density
PEPT1	Oligopeptide transporter 1
CML	Nε-carboxymethyllysine
qRT-PCR	Real time quantitative polymerase chain reaction
ARE	Antioxidant responsive element
WB	Western blot
GAPDH	Glyceraldehyde-3-phosphate dehydrogenase
IOD	Integrated optical density
SDS-PAGE	Sodium dodecyl sulfate–polyacrylamide gel electrophoresis
