# Supplementary figures and images for: Different Regulatory Effects of Heated Products and Maillard Reaction Products of Half-Fin Anchovy Hydrolysates on Intestinal Antioxidant Defense in Healthy Animals
Source: Int J Mol Sci. 2023 Jan 25;24(3):2355. doi: 10.3390/ijms24032355 (PMC9917108; doi:10.3390/ijms24032355)

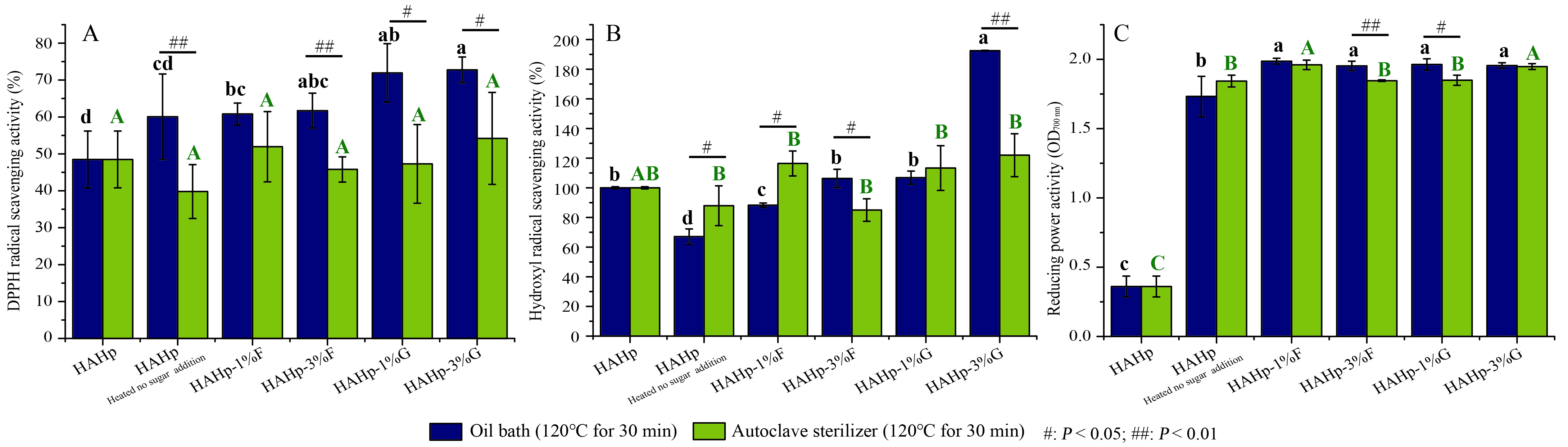

Supplement: Supplementary file 1 [file ijms-24-02355-s001.zip › Figure S1.jpg]

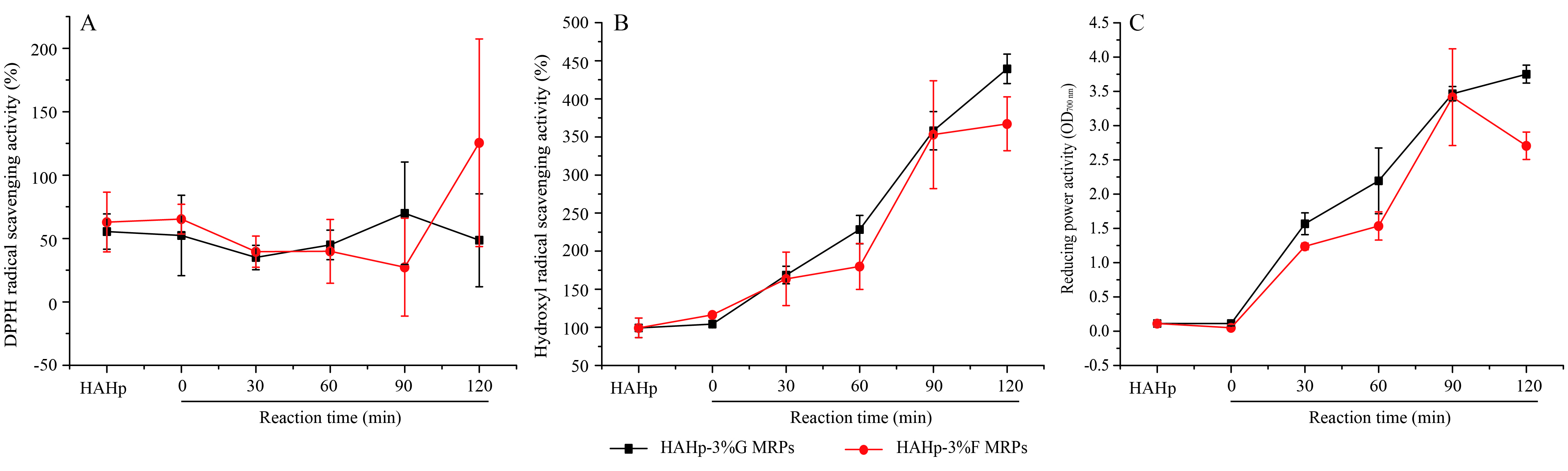

Supplement: Supplementary file 1 [file ijms-24-02355-s001.zip › Figure S2.jpg]
